# Flies from a tertiary hospital in Rwanda carry multidrug-resistant Gram-negative pathogens including extended-spectrum beta-lactamase-producing *E. coli* sequence type 131

**DOI:** 10.1186/s13756-020-0696-y

**Published:** 2020-02-17

**Authors:** Stefan E. Heiden, Mathis S. E. Kurz, Jürgen Bohnert, Claude Bayingana, Jules M. Ndoli, Augustin Sendegeya, Jean Bosco Gahutu, Elias Eger, Frank P. Mockenhaupt, Katharina Schaufler

**Affiliations:** 1 Pharmaceutical Microbiology, Institute of Pharmacy, University of Greifswald, Friedrich-Ludwig-Jahn-Str. 17, 17489 Greifswald, Germany; 2 Charité-Universitätsmedizin Berlin, Berlin, Germany; 3 University Medicine Greifswald, Greifswald, Germany; 4 University of Rwanda, Kigali, Rwanda; 5 University Teaching Hospital of Butare, Butare, Rwanda

**Keywords:** MRGN, Vector flies, Virulence

## Abstract

Multidrug-resistant gram-negative (MRGN) bacteria are a serious threat to global health. We used genomics to study MRGN obtained from houseflies in a tertiary Rwandan hospital. Our analysis revealed a high abundance of different MRGN including *E. coli* pathogenic lineage ST131 suggesting the important role of flies in disseminating highly virulent pathogens in clinical settings and beyond.

## Text

Multidrug-resistant gram-negative (MRGN) bacteria include *Escherichia* (*E.*) *coli*, *Klebsiella* spp., *Enterobacter (E.) cloacae, Acinetobacter* spp., and *Pseudomonas* (*P.*) *aeruginosa*, and others, and cause a variety of severe infections like diarrhea, pneumonia, sepsis, endocarditis and urinary tract infection (UTI). Studies estimate 700.000 fatalities caused by antibiotic-resistant pathogens each year with increasing numbers [1]. In addition to their common occurrence as nosocomial pathogens, MRGN have been frequently found in livestock and the environment. Flies have only recently come into spotlight as carriers of resistant bacteria, and their major route of colonization stems from walking on contaminated surfaces [2]. The detection of antibiotic-resistant *E. coli* from flies captured in a livestock facility was thus unsurprising [3]. Another study has shown that houseflies from hospitals in the UK carried different bacteria resistant to antibiotics [4]. We investigated if houseflies captured in a tertiary hospital in Rwanda carried clinically relevant MRGN pathogens. In African hospital settings, where hygienic conditions may be suboptimal [5], flies might function as underestimated vectors for the distribution of antibiotic-resistant bacteria.

**Fig. 1:**
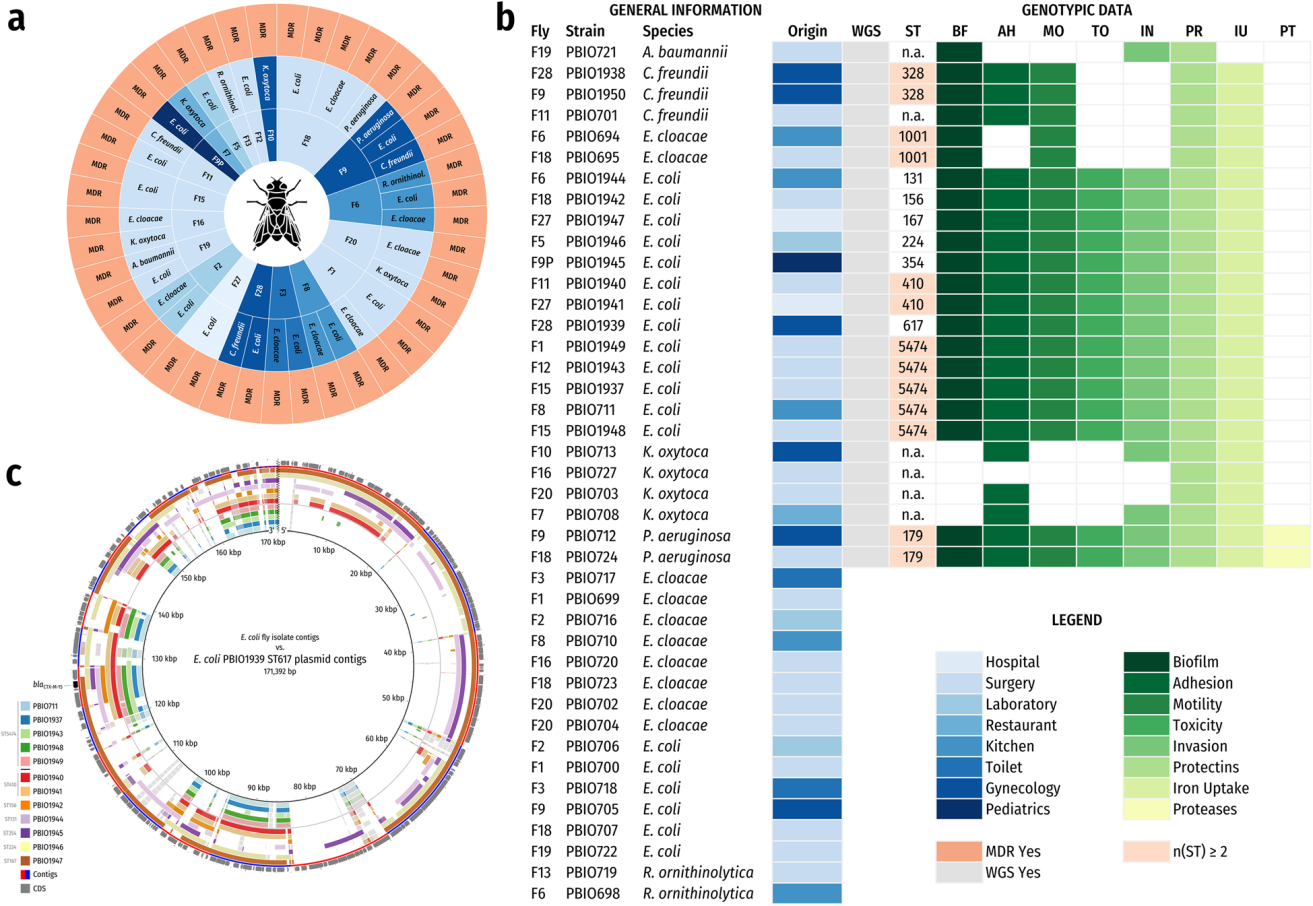
**a**: Overview of all flies carrying MRGN and associated resistance phenotypes (MDR: multidrug-resistant); **b**: Overview of all flies carrying MRGN and associated genotypic data (WGS: whole genome sequencing; ST: sequence type; n.a.: not applicable); **c**: BRIG (Blast Ring Image Generator) comparison of plasmid sequences of all *E. coli* strains with PBIO1939 as reference

We examined 42 flies randomly captured in fly traps within 4 weeks in a tertiary hospital in Rwanda in 2014 [5]. Sampling locations included surgery, gynecologic and other wards (Fig. 1a/b). Because we initially focused on cefotaxime-resistant representatives, bacteria carried by flies were first enriched in tryptic soy broth and then cultured on chromogenic agar (CHROMagar-ESBL, Mast Diagnostica, Germany) supplemented with 2 μg/mL cefotaxime. For “extended-spectrum beta-lactamase (ESBL)-positive” colonies, ESBL and/or ampicillinase (AmpC) production was verified (ESBL-AmpC-Detection Test, Mast Diagnostica [6]), and strains positive for AmpC only were excluded. After preselecting putative strains of *E. coli, Klebsiella* spp*.*, *Enterobacter* spp*., Acinetobacter* spp*., P. aeruginosa, Citrobacter* spp., and *Raoultella* spp., we confirmed the bacterial species using MALDI-TOF (Bruker Daltonics, Germany). Additional phenotypic resistance screening was performed on the VITEK 2 system (bioMérieux, France) and for colistin resistance on 96-well microtiter plates investigating minimal inhibitory concentrations in triplicates. Randomly selected strains (Fig. 1a/b) were whole genome sequenced (WGS) on an Illumina MiSeq/NovaSeq 6000 (Eurofins Genomics Europe Sequencing GmbH, Germany). Raw reads were quality-trimmed, adapter-trimmed and contaminant-filtered using BBDuk from BBTools (https://sourceforge.net/projects/bbmap/files/BBMap_38.41.tar.gz/download). After *de-novo* assembly using shovill/SPAdes (https://github.com/tseemann/shovill/archive/v1.0.4.tar.gz; http://cab.spbu.ru/files/release3.13.1/SPAdes-3.13.1.tar.gz) and Velvet, draft genomes were polished by mapping all trimmed reads back to the contigs with bwa (https://github.com/lh3/bwa/releases/download/v0.7.17/bwa-0.7.17.tar.bz2) and calling variants with Pilon (https://github.com/broadinstitute/pilon/releases/download/v1.23/pilon-1.23.jar). *E. coli* plasmid sequences of PBIO711 and PBIO1939 were manually extracted using similarity searches (BLASTn Megablast) against the NCBI nucleotide collection for visualization in BRIG (Blast Ring Image Generator) (https://sourceforge.net/projects/brig/files/dev/BRIG-0.95-dev.0004.zip/download). Sequence type (ST), antibiotic resistance/virulence gene and single-nucleotide polymorphism (SNP) detection was carried out using mlst, abricate, and snippy (https://github.com/tseemann/mlst/archive/v2.16.1.tar.gz; https://github.com/tseemann/abricate/archive/v0.8.11.tar.gz; https://github.com/tseemann/snippy/archive/v4.4.1.tar.gz). We inferred a core SNP phylogeny for ST5474. Alignments were filtered for recombinations using Gubbins (https://github.com/sanger-pathogens/gubbins/archive/v2.3.4.tar.gz) and core SNPs extracted using snp-sites (1745 sites; https://github.com/sanger-pathogens/snp-sites/archive/v2.4.1.tar.gz). A maximum likelihood tree was inferred with RAxML-NG (https://github.com/amkozlov/raxml-ng/releases/download/0.9.0/raxml-ng_v0.9.0_linux_x86_64.zip) using GTR + G. The best-scoring maximum likelihood tree was midpoint-rooted and visualized in FigTree (https://github.com/rambaut/figtree/releases/download/v1.4.4/FigTree.v1.4.4.zip).

Overall 48% (20/42) of flies carried antibiotic-resistant bacteria. Thirty-six percent (15/42) carried ESBL-producing *E. coli*, 19% (8/42) *E. cloacae*, 9% (4/42) *K. oxytoca,* 7% (3/42) *C. freundii,* 4% (2/42) *R. ornithinolytica,* 4% (2/42) *P. aeruginosa,* and 2% (1/42) *A. baumannii.* Twelve flies (29%) carried more than one antibiotic-resistant bacterial genus of which three (F6, F9 and F18) carried three different pathogens (Fig. 1a/b).

All strains were phenotypically multidrug-resistant and thus termed MRGN (Fig. 1a), however they were not resistant to carbapenems or colistin. WGS revealed carriage of different antimicrobial resistance genes such as *bla*_CTX-M-15,_ *aac* [3]*-IIa,* and *tet*(A)/(B) (Table S1). Eight different STs were observed including ST131 and ST410 (Fig. 1b). Interestingly, these represent international high-risk clonal lineages [7, 8], which combine antimicrobial resistance with high-level virulence. The ST131 strain harbored ten resistance genes and 31 virulence-associated genes including the *pap* operon linked to UTI [9] (Table S1).

In addition, we observed five *E. coli* strains of ST5474, which is a ST recently associated with enterotoxigenic *E. coli* (ETEC) causing diarrhea [10]. This might point towards fly pollution through stool-contaminated surfaces, possibly through a common source. However, note that we did not detect the ETEC-defining heat-labile and/or heat-stable toxins. Our phylogenetic analysis suggested clonality among our five ST5474 strains (1–9 SNPs/aligned Mbp), and similarity to five publicly available ST5474 genomes (178–560 SNPs/aligned Mbp) (Figure S2).

Three *E. coli* strains (PBIO1939, PBIO1940 and PBIO1941), which did not only originate from individual flies captured in different wards but belonged to two different clonal lineages (ST410 and ST617), carried similar resistance genes (Table S1), however they differed in their overall plasmid content (Fig. 1c).

The two *P. aeruginosa* genomes contained several previously described virulence features mandatory for severe invasive infections including flagella, the type III secretion system, type IV pili, as well as toxins and proteases. The *A. baumannii* genome carried virulence genes associated with serum survival and invasion (phospholipase PLC) (Table S1). Overall, all analyzed genomes showed high virulence potentials (Fig. 1b).

Our results demonstrate that half of the flies in this tertiary hospital in Rwanda carried virulent MRGN pathogens including the pathogenic clonal *E. coli* lineage ST131. High pre-admission and even higher discharge rates at this facility [5] may suggest that a) patients and caregivers were the source of MRGN for the flies and b) that flies play a role in the transmission of antimicrobial-resistant pathogens within clinics and in mirroring the burden of antimicrobial resistance [4] at that time. Even though the actual transmission of MRGN bacteria through flies to humans awaits verification, respective modelling results point strongly into this direction [11].

## Supplementary information

**Additional file 1: Table S1.** Results based on whole genome sequence analysis. Abbreviations: BLA: beta-lactams (incl. ampicillin, piperacillin, cefuroxime, cefpodoxime, cefotaxime, ceftazidime); GEN: gentamicin; CIP: ciprofloxacin/moxifloxacin; SXT: sulfamethoxazole-trimethoprim; TET: tetracycline; ST: sequence type; resistance, virulence and plasmid genes are based on the abricate (https://github.com/tseemann/abricate) abbreviations using the databases Resfinder, ARG-ANNOT, CARD, NCBI Bacterial Antimicrobial Resistance Reference Gene Database, PlasmidFinder, VFDB, and Ecoli_VF.

**Additional file 2: Figure S2.** Phylogenomic tree of five *E. coli* sequence type (ST) 5474 fly isolates (strain names colored according to Fig. 1c) and publicaly available WGS data of five ST5474 strains (raw read accession nos.; black).

## Data Availability

The data for this study have been deposited in the European Nucleotide Archive (ENA) at EMBL-EBI under accession number PRJEB36565 (https://www.ebi.ac.uk/ena/data/view/PRJEB36565).

## Abbreviations

ESBL: Extended-spectrum beta-lactamase
ETEC: Enterotoxigenic *E. coli*
MRGN: Multidrug-resistant gram-negative
SNP: Single-nucleotide polymorphism
ST: Sequence type
UTI: Urinary tract infection
WGS: Whole genome sequencing
