# Supplementary figures and images for: Flies from a tertiary hospital in Rwanda carry multidrug-resistant Gram-negative pathogens including extended-spectrum beta-lactamase-producing E. coli sequence type 131
Source: Antimicrob Resist Infect Control. 2020 Feb 17;9:34. doi: 10.1186/s13756-020-0696-y (PMC7026959; doi:10.1186/s13756-020-0696-y)

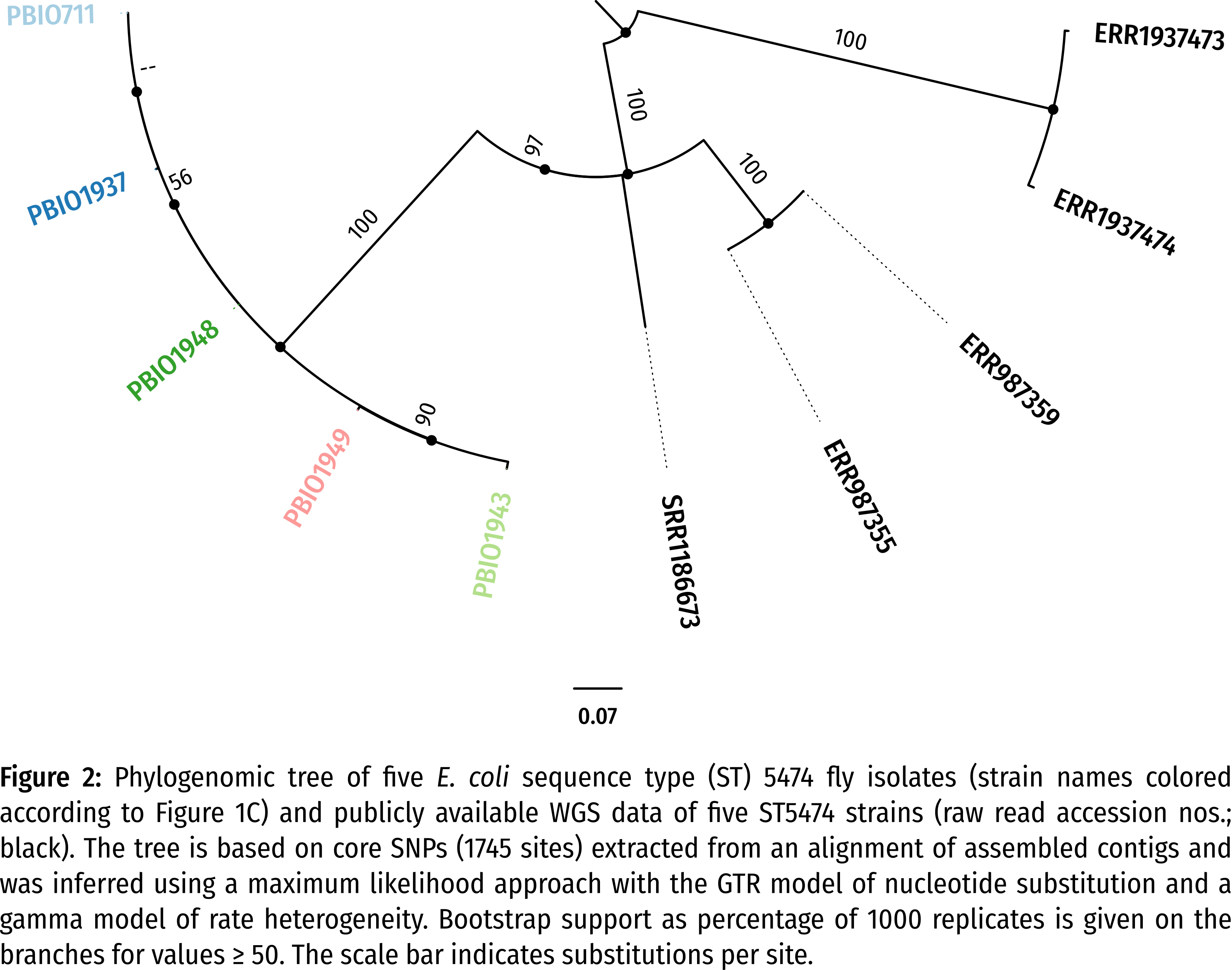

Supplement: Supplementary file 2 — Additional file 2: Figure S2. Phylogenomic tree of five E. coli sequence type (ST) 5474 fly isolates (strain names colored according to Fig. 1c) and publicaly available WGS data of five ST5474 strains (raw read accession nos.; black). [file 13756_2020_696_MOESM2_ESM.jpg]
